# Palliative care nursing roles in the acute inpatient setting: A scoping review

**DOI:** 10.1017/S1478951525100795

**Published:** 2025-11-12

**Authors:** Rachel Heda-Joy Forrest, Louise O’Connell

**Affiliations:** 1 Te Kura Tāpuhi – School of Nursing, Te Kura Hauora Tangata – College of Health, Te Kunenga Ki Pūrehuroa – Massey University, Wellington, New Zealand; 2 Clinical Nurse Specialist Kaimahi Matanga Nēhi, Palliative Care Advisory Service – Southern, Dunedin Hospital, Dunedin, New Zealand

**Keywords:** Holistic care, care-coordination, inpatient, palliative, symptom management

## Abstract

**Aim:**

This study aimed to explore the many roles of palliative care (PC) nurses in addressing the needs of patients with life-limiting illnesses in the acute inpatient setting in New Zealand.

**Methods:**

A scoping review was undertaken, informed by the Joanna Briggs Institute guidelines and utilizing the Preferred Reporting Items for Systematic Reviews and Meta-Analyses (PRISMA) framework. In December 2024, a comprehensive search using Pubmed, Scopus, and CINAHL was conducted for peer-reviewed articles published in English between the years 2014 and 2024, with full text available, that focused on PC nursing in the acute setting in New Zealand, Australia, Canada, Ireland, or the United Kingdom. These countries were chosen because their health care systems are similar. Citation searches were undertaken. Grey literature from New Zealand hospitals was also searched.

**Results:**

After selection, 25 pieces of literature were eligible for the review. Nine key areas were found where PC nurses and teams play key roles in the ongoing management of patients and their coordination of care within the last months to last days of life in the acute inpatient setting. The areas were care-coordination, communication facilitation, decision-making, goals and expectations, discharge planning, physical symptom management, holistic symptom management, finances, environment, education, and rapid review. The findings suggest that PC nurses in the acute setting are no longer involved in single episodes of symptom management and ward-based end-of-life nursing, but are responsible for multiple facets of care, facilitated across several different services.

**Significance of results:**

Understanding the complex roles involved in PC nursing can impact the health care outcomes of patients with a life-limiting illness. The scoping review can help inform future staffing requirements and the skill mix and knowledge levels required to provide timely and appropriate PC in the acute environment in New Zealand hospitals.

## Introduction

Palliative care (PC) is an integral part of health care, addressing the individual needs of patients with life-limiting illnesses, whether they require symptom management and support earlier in their illness trajectory or are in the final stages of life (El Majzoub et al. 2019). The acute hospital setting plays a significant role in end-of-life (EOL) care, with approximately 30% of deaths in Aotearoa New Zealand, a figure similar to global reports of 30–51% (Gott et al. 2017; Donnelly et al. 2018; Australian Bureau of Statistics 2021; Statistics Canada 2023). The increasing global population over 60 years old, projected to reach one-fifth of the world’s total by 2050, coupled with the rising prevalence of comorbidities and complex medical conditions, highlights the escalating need for PC services within hospitals (Waller et al. 2020). PC nurses, with their specialized training and presence across all wards, are uniquely positioned to provide holistic care to patients, families, and medical teams, in addition to traditional bedside nursing. The expanding role of PC nurses, the growing complexity of patient needs, and the projected increase in demand necessitate a critical evaluation of the current PC workforce and a proactive approach to future workforce planning. This scoping review explores the breadth of PC nursing roles in the acute inpatient setting by examining published literature about supportive roles in PC within New Zealand, Australia, Canada, the United Kingdom, and Ireland.

## Methods

This scoping review systematically mapped the existing literature to provide a broad understanding of PC nursing roles in acute hospital settings. The review adhered to the Joanna Briggs Institute (JBI) scoping review guidelines and utilized the Preferred Reporting Items for Systematic Reviews and Meta-Analyses for scoping reviews (PRISMA-ScR) checklist and flow diagram (Tricco et al. 2018; Peters et al. 2020). Eligibility criteria were defined before the literature search (Table 1). The review focused on English-language, peer-reviewed articles published between January 2014 and December 2024, exploring the roles and responsibilities of PC nurses in acute inpatient hospital settings within New Zealand, Australia, the UK, Ireland, and Canada. Studies focusing solely on patient outcomes or interventions not directly related to nursing roles or settings outside of acute care were excluded. The search strategy was developed with a Massey University Health Librarian (Table 2). CINAHL, Scopus, and PubMed were searched, along with citation searching and Google Scholar. Search terms included keywords and combinations related to PC nurses, roles, responsibilities, acute settings, and inpatient wards. Gray literature from New Zealand hospitals was also searched. The retrieved articles and gray literature were entered into an Excel® spreadsheet, and duplicates were removed. Relevant data from the articles were charted using a table for consistency (Pollock et al. 2021).

**Table 1.** Eligibility criteria for literature screening before review

Category	Inclusion criteria	Exclusion criteria
Language	English language articles	Non-English language articles
Article types and sources	Open access, primary research, opinion pieces, and reviews from peer-reviewed journals. Grey literature from hospitals.	Restricted access, non-peer-reviewed articles, grey literature from other sources, articles on pre-publication platforms
Publication range	January 2014–December 2024	Out of the specified inclusion range
Population of interest	Palliative care nurses working in acute hospital settings in New Zealand, Australia, the United Kingdom, Ireland, and Canada, reflecting similar public health systems	Palliative care nurses who are working in acute hospital settings in other countries. Other health care professionals, patients, or family members, regardless of country
Concept/topic of interest	Studies that mention the roles and responsibilities of palliative care nurses, including tasks related to symptom management, care coordination, communication, and collaboration	Studies focusing solely on patient outcomes, interventions not directly related to the roles and responsibilities of palliative care nurses
Context/clinical settings	Acute settings within public hospitals, including medical, surgical, and emergency departments, and intensive care units.	Community settings, residential care, long-term care, rehabilitation facilities, hospice care

**Table 2.** Search queries

Database	Search query
CINAHL	(MH “Palliative Care”) OR (MH “Terminal Care”) OR (MH “Hospice Care”) OR palliative* OR “end of life” OR terminal* OR hospice* AND (MH “Nurses +”) OR nurs* AND (MH “Role” OR “Roles” OR “Responsibilities” OR “Tasks”) AND (MH “Emergency Service”) OR “emergency service*” OR “acute setting*” OR “acute care*” OR “emergency room*” OR “emergency department*” OR “inpatient ward*”
Scopus	palliative* OR “end of life” OR terminal* OR hospice* AND nurs* AND (“role” OR “roles” OR “responsibilities” OR “tasks”) AND (“emergency service*” OR “acute setting*” OR “acute care*” OR “emergency room*” OR “emergency department*” OR “inpatient ward*”)
	Out of the specified inclusion range
PubMed	(palliative care OR “palliative care” OR terminal care OR “terminal care” OR hospice care OR “hospice care” OR “end of life care”) AND (nurse OR nurs*) AND (role OR roles OR responsibilities OR tasks) AND (hospital emergency service OR “emergency service” OR “acute setting*” OR “acute care*” OR “emergency room*” OR “emergency department*” OR “inpatient ward*”)

## Results

### Characteristics of included studies

**Figure 1.**
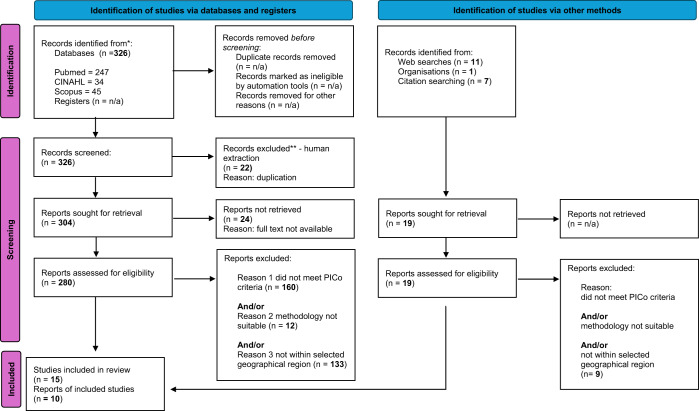
PRISMA flow diagram.

The search and screening outcomes are presented in the PRISMA flow diagram shown in Figure 1. The literature was primarily qualitative and from the UK (Table 3). These articles explored various aspects of PC in acute hospital settings, with a focus on improving patient outcomes and experiences.

**Table 3.** A matrix of country versus type of study

Geographic location	Review articles	Quantitative articles	Qualitative articles	Mixed methods articles	Other
**Multiple countries**	Bajwah et al. (2020), Piazza and Drury (2023), Xiong et al. (2023)				
**New Zealand**	Robinson et al. (2020)	Coombs et al. (2016)	Killackey et al. (2020)	Coombs et al. (2015)	Southern District Health Board Service Plan (2018−2020)
**Australia**	Virdun et al. (2016)	Sweeny et al. (2024)	Fox et al. (2016), Shepherd et al. (2021), Omoya et al. (2024)		
**UK**		Bone et al. (2019)	Bailey et al. (2016), O’Brien et al. (2019), Paes et al. (2018), Chen et al. (2019), Redwood et al. (2020)	Bone et al. (2021)	
**Ireland**	Nevin et al. (2020)		Donnelly et al. (2018)		
**Canada**		Quinn et al. (2020)	Morey et al. (2021), Reid et al. (2023)		

Table 4 presents the characteristics of the 25 articles. The key commonalities and differences were identified from the data extracted. Several studies highlighted the need for early identification and involvement of PC specialists to improve patient outcomes, symptom management, and discharge planning (Coombs et al. 2016; Bone et al. 2019, 2021; Chen et al. 2019; Killackey et al. 2020; Quinn et al. 2020; Sweeny et al. 2024). Effective communication and coordination among health care professionals, patients, and families were also identified as being crucial for providing quality PC (Fox et al. 2016; Nevin et al. 2020; Redwood et al. 2020; Morey et al. 2021; Shepherd et al. 2021). Several studies emphasized the importance of addressing the physical, psychological, social, spiritual, and cultural needs of patients and their families at the EOL (Coombs et al. 2015; Virdun et al. 2016; Donnelly et al. 2018; O’Brien et al. 2019; Robinson et al. 2020; Reid et al. 2023). Other articles identify various barriers to providing optimal PC in acute care settings, including organizational and systemic issues, lack of resources, and communication difficulties (Southern District Health Board 2018; Nevin et al. 2020; Shepherd et al. 2021; Xiong et al. 2023; Omoya et al. 2024).

**Table 4.** Eligible articles’ characteristics

Full APA citation	Country and palliative care (PC) nursing focus	Area of interest	Context	Methodology	Key findings
Bailey, C., Hewison, A., Karasouli, E., Staniszewska, S., & Munday, D. (2016). Hospital care following emergency admission: a critical incident case study of the experiences of patients with advanced lung cancer and Chronic Obstructive Pulmonary Disease. *Journal of Clinical Nursing, 25(15−16)*, 2168−2179. https://doi.org/10.1111/jocn.13170	United Kingdom: Patients with advanced lung disease and chronic obstructive pulmonary disorder (COPD).	Experiences of care received after initial emergency care was stabilized.	Emergency department (ED), followed by acute medical and respiratory wards.	Qualitative critical incident study.	Care following an ED presentation can potentially be improved in the acute setting. Care planning and involving family and the community should be a priority for advanced lung disease patients so they can remain independent and in the community for longer.
Bajwah, S., Oluyase, A. O., Yi, D., Gao, W., Evans, C. J., Grande, G., Todd, C., Costantini, M., Murtagh, F. E., & Higginson, I. J. (2020). The effectiveness and cost-effectiveness of hospital-based specialist palliative care for adults with advanced illness and their caregivers. *The Cochrane Database of Systematic Reviews, 9(9)*, Article CD012780. https://doi.org/10.1002/14651858.CD012780.pub2	Multiple countries: Adults with advanced illness.	To investigate hospital-based specialist PC.	Inpatient settings.	Quantitative: Systematic review of Randomized Controlled Trials.	Minimal evidence that hospital specialist PC has a benefit to patients and their unpaid caregivers. There are small benefits for symptom management and discharge destination, but overall, more research is needed to measure outcomes around symptom management, place of care, caregiver outcomes, and cost-effectiveness of the team.
Bone, A. E., Evans, C. J., Henson, L. A., Gao, W., & Higginson, I. J. (2019). Patterns of emergency department attendance among older people in the last three months of life and factors associated with frequent attendance: a mortality follow-back survey. *Age & Ageing, 48(5),* 680−687. https://doi.org/10.1093/ageing/afz043	United Kingdom: Older people in the last 3 months of life.	Continuity of care at end of life (EOL) – uninterrupted by emergency visits.	EDs within acute hospitals.	Quantitative follow-up surveys of ED attendance.	Older people with multiple comorbidities experience fewer visits to EDs towards EOL, when care coordination is improved, either by a key health professional or a similar team, to oversee care.
Bone, A. E., Evans, C. J., Henson, L. A., Etkind, S. N., & Higginson, I. J. (2021). Influences on emergency department attendance among frail older people with deteriorating health: a multicentre prospective cohort study. *Public Health, 194, 4−10*. https://doi.org/10.1016/j.puhe.2021.02.031	United Kingdom: Frail older people with deteriorating health.	Identifying patients requiring PC during admission and for discharge planning.	Hospital inpatient settings.	Convergent mixed methods design: Qualitative narrative study and quantitative inpatient clinical data	Older people have several missed opportunities to engage with PC services for discharge support, in order to optimize recovery with or without ongoing care.
Chen, H., Walabyeki, J., Johnson, M., Boland, E., Seymour, J., & Macleod, U. (2019). An integrated understanding of the complex drivers of emergency presentations and admissions in cancer patients: Qualitative modelling of secondary-care health professionals’ experiences and views. *PloS one, 14(5)*, Article e0216430. https://doi.org/10.1371/journal.pone.0216430	United Kingdom: New referrals to inpatient PC from ED with advanced cancer.	Planning service follow-up with PC and multi-disciplinary teams (MDT) to support patients and reduce ED admissions.	ED and ward setting.	Qualitative study of health care professionals’ experiences and perceptions around ED admissions.	Specialist PC advice could be sought earlier in presentation for discussion of increased treatment versus PC approach; communication skills during difficult conversations
Coombs, M., Fulbrook, P., Donovan, S., Tester, R., & deVries, K. (2015a). Certainty and uncertainty about end of life care nursing practices in New Zealand Intensive Care Units: A mixed methods study. *Australian Critical Care, 28(2),* 82−86. https://doi.org/10.1016/j.aucc.2015.03.002	New Zealand: Nursing frameworks and practice in intensive care units (ICUs).	Providing holistic care at EOL in highly charged settings.	ICUs	Mixed method – cross-sectional survey followed by focus groups.	Diverse opinions and understandings were held about some EOL care nursing practices. Most ICUs provide culturally sensitive and appropriate EOF care in New Zealand, including open conversations with whānau (family) and alleviated confusion around practices that may be distressing.
Coombs, M., Nelson, K., Psirides, A., Suter, N., & Pedersen, A. (2016). Characteristics and dying trajectories of adult hospital patients from acute care wards who die following review by the rapid response team. *Anaesthesia and Intensive Care, 44(2),* 262−269. https://doi.org/10.1177/0310057X1604400213	New Zealand: Rapid response teams’ experiences with palliative patients.	Acute setting assessments and handing over care to PC teams	All acute settings in hospitals.	Quantitative: Retrospective descriptive cohort study.	There is a need to understand what an acute Rapid response team could offer a dying patient and when they should be transferred to the care of the PC team.
Donnelly, S., Prizeman, G., Ó Coimín, D., Korn, B., & Hynes, G. (2018). Voices that matter: end-of-life care in two acute hospitals from the perspective of bereaved relatives. *BMC Palliative Care, 17(1)*, Article 117. https://doi.org/10.1186/s12904-018-0365-6	Ireland: Dying patients and their families.	Assessment and measure of the quality of care at EOL.	Two acute hospitals.	Quantitative descriptive post-bereavement study.	It is important to seek feedback from families of deceased patients to ascertain the levels of care given at EOL, and improve on their quality.
Fox, J., Windsor, C., Connell, S., & Yates, P. (2016). The positioning of palliative care in acute care: A multiperspective qualitative study in the context of metastatic melanoma. *Palliative & Supportive Care, 14(3)*, 259−268. https://doi.org/10.1017/S1478951515000917	Australia: Palliative patients in the acute phase of metastatic melanoma.	Negotiating the transition to PC.	Variety of acute care settings.	Qualitative, theoretical framework, with grounded theory to interview patients, carers, and health care professionals.	There is uncertainty around the scope of practice in the transition to PC. Findings can be generalized more broadly to other diseases, that there is a need to achieve coherency of care within very specialized health systems. More clarity is required to define “Palliative Care” and its role within the acute health care system.
Killackey, T., Lovrics, E., Saunders, S., & Isenberg, S. R. (2020). Palliative care transitions from acute care to community-based care: A qualitative systematic review of the experiences and perspectives of health care providers. *Palliative Medicine, 34(10),* 1316−1331. https://doi.org/10.1177/0269216320947601	New Zealand: Patients transitioning from acute settings to community for EOL care.	Coordinating the transition to home.	Hospital-to-home settings	Qualitative systematic review of experiences.	Multiple different and complex roles are experienced by health care providers in preparing for a patient to be discharged home from the hospital, for EOL care, including goal setting, discharge planning, education, and expectations.
Morey, T., Scott, M., Saunders, S., Varenbut, J., Howard, M., Tanuseputro, P., Webber, C., Killackey, T., Wentlandt, K., Zimmermann, C., Bernstein, M., Ernecoff, N., Hsu, A., & Isenberg, S. (2021). Transitioning from hospital to palliative care at home: Patient and caregiver perceptions of continuity of care. *Journal of Pain and Symptom Management, 62(2),* 233−241. https://doi.org/10.1016/j.jpainsymman.2020.12.019	Canada: Patients experiencing EOL.	Transition from acute to PC both in acute setting and transfer to PC at home.	Hospital wards.	Qualitative longitudinal design, semi-structured interviews with patients before and after the transition from hospital to PC.	Most patients interviewed experienced continuity of care from hospital to home, through information exchange (between hospital providers), consistent treatments (in hospital) to consistency in providers (home). Communication and reliable information transfer were key components to successful discharge from acute care to home.
Nevin, M., Hynes, G., & Smith, V. (2020). Healthcare providers’ views and experiences of non-specialist palliative care in hospitals: A qualitative systematic review and thematic synthesis. *Palliative Medicine, 34(5)*, 605−618. https://doi.org/10.1177/0269216319899335	Ireland: Patients in hospital with PC needs.	Non-specialist PC in assisting and supporting decision-making.	Hospitals.	Qualitative systematic review and thematic synthesis.	There are multiple barriers to quality PC in acute care. Culture, organization of acute care, and dysfunctional MDTs are just some of the issues leading to poor patient experiences. Communication between doctors and nurses can cause confusion and poor decision-making when deciding whether or not to withdraw cares.
O’Brien, M. R., Kinloch, K., Groves, K. E., & Jack, B. A. (2019). Meeting patients’ spiritual needs during end-of-life care: A qualitative study of nurses’ and healthcare professionals’ perceptions of spiritual care training. *Journal of Clinical Nursing, 28(1/2)*, 182−189. https://doi.org/10.1111/jocn.14648	United Kingdom: Patients at EOL.	Recognizing, assessing and addressing the spiritual issues of PC patients at EOL.	Acute hospitals, Hospice, residential care and community.	Qualitative – semi-structured interviews with nurses and health care professionals, data subject to thematic analysis.	Patients with unmet spiritual needs at EOL, are vulnerable and at risk of poorer psychological outcomes and diminished quality of life. There is a potential for nurses and health care professionals to avoid conversations and support due to perceived lack of experience in spiritual matters.
Omoya, O., De Bellis, A., & Breaden, K. (2024). “Caught in the middle” – emergency doctors and nurses’ experiences of ethical dilemmas in end of life care: A qualitative study*. International Emergency Nursing, 77*. https://doi.org/10.1016/j.ienj.2024.101535	Australia: Patients at EOL.	Ethical decision-making in EOL cares.	Acute ED.	Qualitative interpretive design.	EOL care in EDs can raise ethical dilemmas for nurses and doctors. Understanding the complexities of caring for dying patients in the acute setting is important to further work on serious decision-making discussions, having open conversations about dying, and making timely referrals to PC teams.
Paes, P., Ellershaw, J., Khodabukus, A., & O’Brien, B. (2018). Palliative care in acute hospitals – a new vision. *Future Healthcare Journal, 5(1),* 15−20. https://doi.org/10.7861/futurehosp.5-1-15	United Kingdom: Palliative inpatients.	Understanding service models in PC	Acute hospital.	Qualitative review of PC Unit model.	New service model puts forward the proposal that PC Units are developed to merge with specialist PC teams in the hospital to create a more efficient and patient-centered service.
Piazza, M., & Drury, A. (2023). An integrative review of adult cancer patients’ experiences of nursing telephone and virtual triage systems for symptom management. *European Journal of Oncology Nursing, 67,* Article 102428. https://doi.org/10.1016/j.ejon.2023.102428	Multiple countries: Adult cancer patients, 2 studies palliative at baseline.	Supportive care.	Telephone and virtual triaging from hospital base.	Qualitative – Integrated review of patient experiences.	Significant evidence showing the impact of nurse-led interventions and specialist nursing roles on symptom management and supportive care outcomes for oncology patients, both for curative and palliative intent.
Quinn, K. L., Stukel, T., Stall, N. M., Huang, A., Isenberg, S., Tanuseputro, P., Goldman, R., Cram, P., Kavalieratos, D., Detsky, A. S., & Bell, C. M. (2020). Association between palliative care and healthcare outcomes among adults with terminal non-cancer illness: population based matched cohort study. *BMJ (Clinical research ed.), 370,* Article m2257 https://doi.org/10.1136/bmj.m2257	Canada: Terminal non-cancer patients.	Improve quality of life at/for discharge.	Across all settings including inpatient.	Quantitative population-based cohort study.	Increasing access to PC through sustained investment in training and model revisions could improve EOL care and decrease hospital re-admissions.
Redwood, S., Simmonds, B., Fox, F., et al. (2020). Consequences of “conversations not had”: insights into failures in communication affecting delays in hospital discharge for older people living with frailty. *Journal of Health Services Research & Policy, 25(4),* 213−219. https://doi.org/10.1177/1355819619898229	United Kingdom: Acutely unwell older people.	Skilled coordination and organization of patient discharges	Hospital.	Semi-structured interviews with health care professionals in hospital setting.	Important conversations about EOL care are often not initiated, including treatment limits and resuscitation status. The topic of death and dying was not raised at the appropriate times, affecting ongoing quality of life, and becomes a barrier to a humane and compassionate dying experience.
Reid, J. C., Dennis, B., Hoad, N., Clarke, F., Hanmiah, R., Vegas, D. B., Boyle, A., Toledo, F., Rudkowski, J. C., Soth, M., Heels-Ansdell, D., Cheung, A., Willison, K., Neville, T. H., Cheung, J., Woods, A., & Cook, D. (2023). Enhancing end of life care on general internal medical wards: the 3 Wishes Project. *BMC Palliative Care, 22(1),* Article 11. https://doi.org/10.1186/s12904-023-01133-4	Canada: EOL care patients on internal medical wards.	Develop and initiate personalized, compassionate care to dying patients and families.	Inpatient settings.	Qualitative, phased multicomponent 2-year cohort study.	Facilitating connections, providing care to families, and personalizing the environment were the most common wishes of participants.
Robinson, J., Moeke, M. T., Parr, J., Slark, J., Black, S., Williams, L., & Gott, M. (2020). Optimising compassionate nursing care at the end of life in hospital settings. *Journal of Clinical Nursing, 29(11−12),* 1788−1796. https://doi.org/10.1111/jocn.15050	New Zealand: EOL care in hospital settings.	To optimize compassion in nursing cares at EOL.	Hospital	Review/Qualitative	Looking at health care in dying through a bicultural lens adds value for all New Zealanders and can be generalized to other similar populations globally.
Shepherd, J., Waller, A., Sanson-Fisher, R., Clark, K., & Ball, J. (2021). Barriers to the provision of optimal care to dying patients in hospital: a cross-sectional study of nurses’ perceptions. *Australian Journal of Advanced Nursing, 38(3),* 14−24. https://doi.org/10.37464/2020.383.315	Australia: Dying patients.	Health care provision at EOL.	Hospital.	Qualitative cross-sectional survey of nurse perceptions.	Optimal EOL care is made challenging by patient, family, provider and health-system–related barriers. A coordinated approach to the provision of EOL care is the key to success.
Southern District Health Board Service Plan Palliative Care Advisory Service 2018−2020	New Zealand: PC patients.	Improve access to PC, coordinate symptom management and discharge planning.	Acute Hospital setting.	Consumer and provider review of current service model.	Several challenges exist with the current specialist model of care, including public health perspectives, integration and management across systems, and capacity and demand (funding). It is important to plan for the future to ensure the success of the team in delivering an exceptional level of care to patients dying in Southern District.
Sweeny, A. L., Alsaba, N., May, K., Huang, Y.-L., Ranse, J., Burrows, E., Crilly, J., Grealish, L., Broadbent, A., Sunny, L., Khatri, M., Ranse, K., Denny, K. J., & Lukin, B. (2024). End-of-life care: A retrospective cohort study of older people who died within 48 hours of presentation to the emergency department. *EMA – Emergency Medicine Australasia, 36(1),* 13−23. https://doi.org/10.1111/1742-6723.14331	Australia: Older people who died in ED.	Symptom management and identifying patient at EOL.	Acute ED.	Qualitative, retrospective cohort study.	It is not always straight forward identifying patients in ED who are at the EOL, and that considering recent admissions, and loss of independence can be signals.
Virdun, C., Luckett, T., Lorenz, K., Davidson, P. M., & Phillips, J. (2017). Dying in the hospital setting: A meta-synthesis identifying the elements of end-of-life care that patients and their families describe as being important. *Palliative Medicine, 31(7),* 587−601. https://doi.org/10.1177/0269216316673547	Australia: Patients dying in hospital.	Consumer feelings about what is supportive care at EOL.	Hospital setting.	Meta-synthesis. A systematic search of 10 databases for qualitative studies on dying in hospitals.	Patients and families consistently report that having a specialist PC team to look after their needs in hospital is the most important relationship, they have in the hospital setting. The value of expert care (physical, symptom management), optimal communication, respectful and compassionate care, valued family involvement in care planning and delivery, maintenance of self-identity for patients, environmental privacy for families, ensuring patient safety, supporting patient choices, preparing families for death and providing contact for families after a patient has died, cannot be over-stated.
Xiong, B., Stirling, C., & Martin-Khan, M. (2023). The implementation and impacts of national standards for comprehensive care in acute care hospitals: An integrative review. *International Journal of Nursing Sciences, 10(4),* 425−434. https://doi.org/10.1016/j.ijnss.2023.09.008	Australia, Norway, United Kingdom: Patients at EOL – Australian data.	Reviewed articles from Australia, UK and Norway to investigate comprehensive care approach for standardized care.	Inpatient setting	Integrative review utilizing databases and grey literature.	Difficulties/challenges in implementing EOL care actions as part of a new conceptual model of standards of care

The studies focus on different patient populations, including those with specific diseases (Fox et al. 2016; Bailey et al. 2016; Quinn et al. 2020; Piazza and Drury 2023), older adults (Bone et al. 2019, 2021; Sweeny et al. 2024), and patients in various stages of illness (Bajwah et al. 2020; Morey et al. 2021). The articles investigate a range of interventions, such as care coordination, specialist PC teams, and supportive care, and examine their impact on various outcomes, including symptom management, quality of life, and patient and family satisfaction (Coombs et al. 2015; Bone et al. 2019; Bajwah et al. 2020; Quinn et al. 2020; Piazza and Drury 2023). The articles employ diverse research methodologies (Table 3), including qualitative, quantitative, and mixed-methods approaches, to explore different aspects of PC in acute care (e.g., patient experiences, health care professional perspectives, and service models).

### Synthesis of results

**Figure 2.**
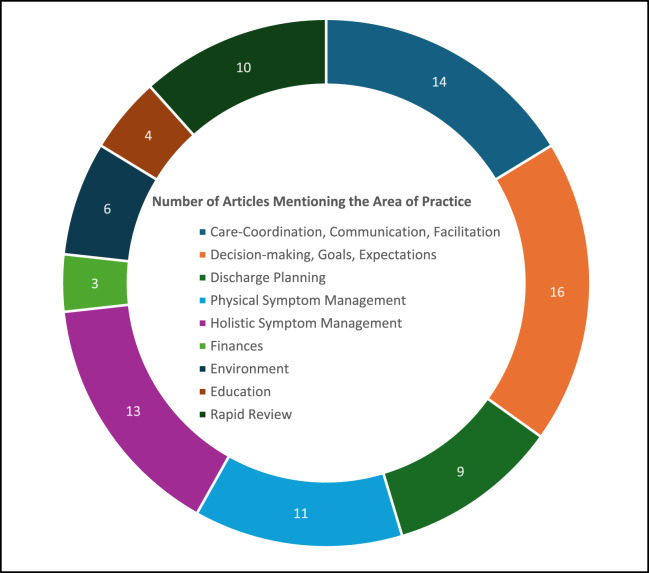
Key areas of palliative care nursing practice identified by the scoping review.

While there was extensive literature on PC nursing, relatively little was focused on the acute care hospital setting. This finding, in itself, reveals the need for research targeted in this area so that a more in-depth understanding of the roles and responsibilities of PC nursing in acute care is gained. The following sections will examine nine key areas identified in the review (Figure 2), reflecting the multifaceted nature of PC nursing in the acute care setting and highlighting the need for further research and policy development to support meeting the needs of hospital patients with life-limiting illnesses and the nurses who care for them.

### Coordination of care, communication, and facilitation

Within the hospital setting, the fragmentation of services was often found to lead to poor communication, discharge planning, and care coordination, requiring specialized PC nursing services to bring it all together (Fox et al. 2016; Bailey et al. 2016; Virdun et al. 2016; Chen et al. 2019; Bajwah et al. 2020; Nevin et al. 2020; Redwood et al. 2020; Shepherd et al. 2021; Reid et al. 2023; Sweeny et al. 2024). The literature highlighted that PC nurses play a crucial role in bridging gaps, coordinating care, facilitating communication in complex situations, ensuring effective transfer of information across settings, and actively participating in patient management planning.

The handing over of patients between different services, for example, from ED to a ward, can be fraught with confusion around the ceiling of care agreed on by patients and families, after assessment by a medical team. High workload volumes in acute areas meant that conversations were not being had by a patient’s treating team, leaving PC services to address with family, why certain interventions were not being administered, and others withdrawn (Fox et al. 2016; Redwood et al. 2020; Shepherd et al. 2021). Three articles noted that the translation of medical information and decisions to patients and families was often left for PC nursing teams to pick up after the initial assessment (Bailey et al. 2016; Virdun et al. 2016; Killackey et al. 2020; Nevin et al. 2020). Effective and compassionate communication was critical to gain a shared understanding of the situation and often relied on the extra time available for PC teams once the patient has been transferred out of an acute assessment area (Bailey et al. 2016; Virdun et al. 2016; Nevin et al. 2020).

PC nursing teams were found to provide support, not only to patients and their caregivers but also to doctors making decisions to withdraw treatment or discontinue futile interventions being mentioned in several articles (Virdun et al. 2016; Bajwah et al. 2020; Nevin et al. 2020; Shepherd et al. 2021). Lack of experience and expertise in junior doctors, low confidence, uncertain prognosis, and fear of saying the wrong thing were barriers to communicating well with EOL patients (Virdun et al. 2016; Bajwah et al. 2020; Nevin et al. 2020; Shepherd et al. 2021). Clinicians could sometimes feel awkward in bringing up the topic of death and dying, and family members may have been too scared to ask questions, leaving the people involved feeling confused and worried about what is happening with the PC nurse being viewed as the “safety net” for these families (Chen et al. 2019; Redwood et al. 2020; Bone et al. 2021; Omoya et al. 2024). On the other hand, if difficult conversations had taken place, the PC nurse’s expertise in communication skills was often vital to facilitate a shared understanding of the situation and for de-escalation (Fox et al. 2016; Chen et al. 2019; Bajwah et al. 2020). The phrase “PC” can be confronting for a patient as they realize treatment may have come to an end, and they are looking to the next stage which involves trying to gain some quality of life for what time remains. Three studies found that by the time patients with a life-limiting illness talk to the PC team, emotions were heightened and complex communication skills were required along with de-escalation of difficult conversations that may have taken place between a consultant and the patient (Fox et al. 2016; Chen et al. 2019; Bajwah et al. 2020). The research identified that effective and compassionate communication was critical to gaining a shared understanding of the situation, and often relied on the extra time available for PC teams once the patient has been transferred out of an acute assessment area.

As well as liaising between doctors and patients, recognizing and coordinating patient care across different inpatient settings was also identified as part of PC nursing services. The research emphasized that PC nurses act as a liaison between various MDTs, such as occupational therapy, physiotherapy, and social work (Bailey et al. 2016; Bajwah et al. 2020; Reid et al. 2023; Sweeny et al. 2024). Facilitating MDT reviews, arranging symptom support services such as domiciliary oxygen, updating primary care or community teams and arranging follow-up services if a patient was discharged home were just some of the areas that needed to be addressed thoughtfully for successful patient outcomes (Bailey et al., 2016; Bajwah et al. 2020; Reid et al. 2023; Sweeny et al. 2024). The studies reviewed highlight the importance of these collaborative efforts in enhancing the overall quality of PC.

Together the literature has revealed that PC nurses play a key role in coordinating care and facilitating communication within the acute hospital setting. PC nurses bridge gaps in service delivery and ensure effective information transfer. The PC nurse’s expertise in communication is particularly crucial in navigating difficult conversations and ensuring a shared understanding among all involved parties to enhance the overall quality of PC and improve patient and family experiences.

### Decision-making, goals, and expectations

PC nurses were found to contribute to decision-making processes, helping patients and families define goals of care, set and manage expectations, and identify gaps in care. Decision-making around cares was a focus of two studies (Fox et al. 2016; Reid et al. 2023). As part of the patient assessment, PC nurses are well placed to facilitate decisions around optimizing quality of life and the current functional status. In the acute care environment, which can look very different compared to what home might be like, shared decision-making was identified as a strong part of patient-centered care (Fox et al. 2016; Reid et al. 2023). The identification of gaps in care, particularly for patients with non-oncological conditions, and the facilitation of the advance care plan (ACP) discussions were identified as important responsibilities of PC nurses.

Two research papers found the ACP discussion was a task often picked up by PC nurses during inpatient stays (Redwood et al. 2020; Sweeny et al. 2024). The ACP documents the treatments and cares that a patient might want towards the EOL, and those interventions they would prefer to avoid, and is a key part of goal-setting. Creating or updating ACPs not only helped to identify patients nearing the EOL and prepared their caregivers for what was coming up, but more importantly, the presence of the ACP was useful in avoiding inappropriate referrals and admissions which could result in further functional decline (Redwood et al. 2020; Sweeny et al. 2024). These research articles found that many times, it was the PC team that alerted the treating clinician that an ACP was in place to guide the patient’s preferences of care.

Goals of care was an area that required more focus and consideration, not only in the broad sense of what is appropriate but also what the patient has defined as acceptable. PC nurses spend considerable time at the bedside, discussing imminent and future goals with patients and families – an area with many expectations from the patient and family. This facet of assessment was mentioned in most of the articles, however, more specifically discussed in six of them (Fox et al. 2016; Chen et al. 2019; Killackey et al. 2020; Nevin et al. 2020; Reid et al. 2023; Shepherd et al. 2021; Xiong et al. 2023). Helping to identify and achieve goals such as getting out of the hospital, staying away from ED, identifying clinical risks and preferences for care, working out how to align clinicians, patient’s and families’ ideas of EOL, finding opportunities for personal growth with limited time, goals for dying well and choosing where to die, were all areas where there was an expectation of PC nurse involvement (Fox et al. 2016; Chen et al. 2019; Nevin et al. 2020; Reid et al. 2021; Shepherd et al. 2021; Xiong et al. 2023). Not only were these goals discussed around the acute inpatient setting, but PC teams were required to broaden this discussion to how life will look in the community, as part of planning for discharge.

Identifying gaps in care was discussed in five studies (Bailey et al. 2016; Bone et al. 2019, 2021; Quinn et al. 2020; Redwood et al. 2020). Non-oncological disease trajectories can be drawn out over several years, in the case of heart failure, chronic obstructive pulmonary disease (COPD) or renal failure. Not only are PC nurses expected to identify the gaps found in the acute system, the studies indicated that education of the patient by PC nursing teams was crucial for living well and staying out of hospital, especially in the context of unlikelihood of hospice community programme admission (Bailey et al. 2016; Bone et al. 2019, 2021; Quinn et al. 2020; Redwood et al. 2020).

The research also highlights that PC nurses play a role in prognosticating illness, a task that can be challenging due to factors such as clinician inexperience and the unpredictable trajectory of non-oncological diseases (Bailey et al. 2016; Virdun et al. 2016; Bajwah et al. 2020; Nevin et al. 2020; Shepherd et al. 2021). Prognostication, while initially the role for the consultant/lead clinician, has become an unwelcome role for PC nurses, especially toward the imminent EOL as family members want frequent updates on how long their loved-one has left to live. Findings suggested that some of the reasons that prognosis was challenging were clinician inexperience, inability to relate to grieving families, not wanting to deliver sad news, and difficulty in understanding the trajectory of non-oncological diseases (Bailey et al. 2016; Virdun et al. 2016; Bajwah et al. 2020; Nevin et al. 2020; Shepherd et al. 2021).

Collectively, the literature details the pivotal role of PC nurses in facilitating patient- and family-centered care within the acute setting. The research acknowledges the involvement of PC nurses in shared decision-making, assisting patients and families in defining goals of care, setting realistic expectations, and identifying gaps in care. The complexities of prognostication, particularly in non-oncological diseases, and the importance of advance care planning discussions are also emphasized. The literature illustrates that PC nurses play a crucial role in bridging the gap between patient/family expectations and the realities of the acute care environment, advocating for patient autonomy and informed decision-making.

### Discharge planning

Almost half the literature reviewed mentioned discharge planning as a common and expected task of PC nursing teams (Table 4, Figure 2). The research showed the significance of discharge planning in ensuring a smooth transition for patients and their families from the hospital to their preferred care setting, whether it be home, hospice, or another facility, with a seamless and coordinated approach to discharge improving the success of PC. PC nursing teams supported continuity of care in the acute setting by acting as a bridge from hospital to home, having already been involved in goals of care discussions, early referral to primary PC services in the community, and assessment in hospital (inpatient wards and ED) for symptom management (Morey et al. 2021; Sweeny et al. 2024). In turn, effective discharge planning contributed to reduced readmission rates and enabled patients to receive appropriate care in their chosen setting (Fox et al. 2016; Bone et al. 2019; Killackey et al. 2020; Morey et al. 2021; Quinn et al. 2020; Redwood et al. 2020; Sweeny et al. 2024).

Information sharing was a key role PC nurses undertook to expedite complex discharges, provide information about community resources, and facilitate access to necessary equipment and medications. PC nursing teams worked with allied health MDTs to support and facilitate rapid and complex discharges back to the patient’s home, either with district nursing or community hospice support (Paes et al. 2018). Information sharing by PC teams between systems meant that discharge delays were less likely, and readmission rates were reduced (Bone et al. 2019; Redwood et al. 2020). As part of their role, PC nursing teams liaised with community services to help patients stay in their homes or facilities. Bone et al. (2021) found that in the last year of life, many older and frail adults were admitted to the ED for crisis admission, yet they would prefer to remain in their usual environments and have PC support in the community. The research by Coombs et al. (2016) revealed the need for a better understanding of what an acute rapid response team can offer a dying patient and when they should be transferred to the care of the PC team. If in the acute care setting, a referral to PC services occurs, the coordination and early assessment by PC teams meant those with a life-limiting illness were more likely to die at home as preferred, due to the discharge supports in place (Fox et al. 2016; Quinn et al. 2020).

Thus, the PC nurses play important roles in facilitating effective discharge planning, which contributes to reduced readmissions, improved patient and family experiences, and help to fulfil patient preferences for EOL care. Early referrals, information sharing, and collaboration with MDTs ensure continuity of care and support of patients’ choices regarding their preferred place of care and death.

### Physical symptom management

As expected, over half of the articles highlighted symptom management as the most important facet of PC nursing team assessment (O’Connor et al. 2016; Virdun et al. 2016; Bone et al. 2019; Chen et al. 2019; Henson et al. 2020; Morey et al. 2021; Quinn et al. 2020; Redwood et al. 2020; Shepherd et al. 2021; Reid et al. 2023; Sweeny et al. 2024; Xiong et al. 2023). In a review of the need for care coordination and shared goals of care, the combined Australian, Norway, and UK comparative study noted that both symptom management and minimization of harm were the most important components of EOL care (Xiong et al. 2023). Other studies from the UK identified having a PC team nurse to assist during admissions with unstable symptoms as an environmental enabler of health when it came to older people living with frailty and turned the conversations away from diagnosis and life-preserving treatment and more toward quality of life remaining (Bone et al. 2019; Redwood et al. 2020). In another UK study of a PC team that attended ED to manage ED presentations in patients diagnosed with terminal cancer with symptoms of pain, nausea, and constipation, it was found that this relationship between the PC team and ED helped to reduce recurrent admissions, improve patient experiences and increased support at the primary level (Chen et al. 2019). Canadian studies have also found that PC teams delivering symptom management assessment and advice in the hospital setting to both cancer and non-cancer related terminal illness patients enabled several positive health outcomes. Patients experienced reduced hospital admission with fewer symptom-related ED visits, which increased their odds of dying at home as a preference, and for those who remained as inpatients for EOL, the overall dying experience for patients and families was improved (Morey et al. 2021; Quinn et al. 2020; Reid et al. 2023). Similarly, in a meta-synthesis by Virdun et al. (2016) that reviewed 16 articles it was found that symptom management at EOL was one of the most important parts of PC in the hospital setting, according to patient and family. Timely and effective relief of physical symptoms, commencement of syringe drivers, and symptom education and support to families were other aspects discussed across the literature (Fox et al. 2016; Southern District Health Board 2018; Sweeny et al. 2024). One study identified the lack of a PC team in the acute setting to be one of the top 5 barriers in effective EOL care (Shepherd et al. 2021). Therefore, PC nurses play a key role in assessing, treating, and managing physical symptoms, with effective symptom management improving patients’ quality of life and reducing the need for ED visits and further hospital admissions.

### Holistic symptom management

Although only one article had the words “spiritual” in its title, many of the articles, as outlined below, discussed spiritual support, along with other ways to provide holistic care for patients with a very limited life expectancy in the hospital setting. The concept of holistic symptom management extends beyond physical symptoms to encompass the psychological, spiritual, social, and existential needs of patients and their families. PC nurses play a crucial role in addressing these multifaceted needs, recognizing the interconnectedness of these dimensions in the experience of suffering (Fox et al. 2016). However, the construction of PC definitions has created tension within some clinical cultures and ambiguity around what holistic care means for EOL patients, with many considering that symptom management should be predominantly concerned with physical issues (Fox et al. 2016). Nevertheless, provision of emotional and psychological support at EOL and facilitating connections appears to have become defined as part of PC teams’ holistic assessment as well as coordinating these and other support services in the acute setting and that included pre-bereavement and anticipatory grief work; the latter classed as specialist PC interventions (Donnelly et al. 2018; Paes et al. 2018; Bajwah et al. 2020; Bone et al. 2021; Reid et al. 2023).

The literature highlighted that families had an understanding that PC nursing teams would meet all the care needs of their loved ones, particularly making everyone feel comfortable about death and dying, as well as caring for the patient physically, emotionally and/or spiritually, and expressed disappointment and disconnection when this was not the case (Fox et al. 2016; Virdun et al. 2016; Morey et al. 2021). It was noted that attending to the patient’s psychological and existential requirements took time, which was a barrier in the acute setting, meaning that there was a need for this role to be taken up by PC teams (Nevin et al. 2020). Unfortunately, one study revealed that 30% of nurses in PC found spiritual and religious facets of care problematic, in part, because they were untrained in these matters (Shepherd et al. 2021). In addition, O’Brien et al. (2019) highlighted that it was not the main responsibility of the chaplaincy service to recognize spiritual distress but instead all PC staff should be trained to identify spiritual needs and should feel comfortable to provide at least a basic level of spiritual care and engaging the Chaplain services when appropriate. Patients were reportedly appreciative when nurses and health care professionals considered their existential needs (O’Brien et al. 2019). Understanding that spiritual care and bereavement were so important to the overall well-being of the EOL patient, one UK Hospital PC team has dedicated staff to assist with this facet of symptom management (Paes et al. 2018). In NZ, one regional hospital policy document requires the PC team to facilitate access to culturally appropriate services for inpatients in the acute setting and recommends that referrals be made for newly diagnosed oncology patients who may need psychological and social work assistance to the dedicated cancer psychosocial team (Southern District Health Board 2018).

The findings presented in this section highlight the challenges faced by PC nurses in providing comprehensive holistic care within the acute setting, particularly in addressing spiritual and existential needs due to time constraints, lack of training, and role ambiguity. The research has reiterated the importance of recognizing and addressing these multifaceted needs to ensure a truly patient- and family-centered approach to PC. The studies suggest that dedicated staff and training in spiritual care, as well as collaboration with other health care professionals like social workers and chaplains, can enhance the provision of holistic support and improve the overall well-being of patients and their families at the EOL.

### Finances

The financial aspects of PC are also addressed in the literature, with a focus on both institutional and personal finances. PC nurses may be involved in navigating funding streams for resources to support patients at home or in higher-level care facilities (Bailey et al. 2016; Quinn et al. 2020). They may also assist patients and families in addressing financial concerns that can contribute to existential distress, either directly or indirectly through referrals to social workers or psychosocial support teams (Virdun et al. 2016).

### Environment

Creating a supportive and comfortable environment for patients nearing the EOL is another important aspect of PC nursing. The research highlights the challenges of finding appropriate spaces within acute hospital settings that allow for privacy, family involvement, and a sense of peace and dignity (Reid et al. 2023). Locating the most appropriate environment for patients who cannot return home and who have less than six weeks to live, as well as those who are imminently dying has become part of the tasks taken on by a PC team. Two studies highlighted that adequate environments for extended family care and support were deficient in acute hospital wards, which did not have spaces that could be personalized to suit the needs of the patient and family (Reid et al. 2023; Omoya et al. 2024). PC nurses play a key role in advocating for patients’ needs and collaborating with other health care professionals to ensure that the physical environment contributes to their overall well-being (Virdun et al. 2016; Donnelly et al. 2018; Reid et al. 2023).

A key feature in PC assessment is identifying that the patient is deteriorating and requires a side/single room, and the speed of this happening was often dependent on the bed status in the hospital at any one time, and liaising with duties managers and charge nurses while negotiating visiting relatives in tight and public spaces was all part of providing optimal PC (Virdun et al. 2016; Reid et al. 2023). The importance of finding a single room was noted to be the overall key factor in a two-site hospital bereavement study, which influenced the dying experience in the acute setting (Donnelly et al. 2018). This concept of selecting an appropriate environment is discussed in Robinson et al. (2020), as a way to honor our bicultural society in NZ, taking into consideration the needs of Māori during the anticipatory grief period and beyond, as a way to truly show compassion in our nursing care. Another NZ article describing EOL-care nursing practices in intensive care units echoed this idea that finding a culturally appropriate place for whanau to support their dying people was not only important but also aligned with the Treaty of Waitangi to underpin nursing practice (Coombs et al. 2015). However, unlike the previous article, the authors reported that NZ ICUs do this well, along with their framework for holistic care – which was not a finding in most of the other reviews on similar acute settings.

The research findings highlight the critical role of PC nurses in advocating for and creating a supportive environment for patients at the EOL. The challenges of securing appropriate spaces within acute care settings that prioritize privacy, family involvement, and a sense of peace and dignity are evident. Furthermore, the ability of PC nurses to navigate these challenges and collaborate with other health care professionals to optimize the patient’s environment significantly impacts the overall quality of EOL care and the dying experience for both patients and their families.

### Education

The literature emphasized the importance of education to enhance understanding and improve the quality of care for patients with life-limiting illnesses. PC nurses are actively involved in educating patients, families, and other health care professionals about EOL care. Ongoing education to non-palliative-trained staff, as well as patients and families, is vital to ensure improved care of the dying and a better understanding of the process for patients and caregivers. PC nurses provide information about symptom management, advance care planning, and available resources (Donnelly et al. 2018; Southern District Health Board 2018; Killackey et al. 2020; Nevin et al. 2020). The research indicated that patient education by PC nursing teams was highly beneficial for living well and staying out of the hospital, especially in the context of the unlikelihood of hospice admission (Bailey et al. 2016; Bone et al. 2019, 2021; Quinn et al. 2020; Redwood et al. 2020). PC teams were also responsible for teaching junior doctors and visiting nurses during patient rounds to support their practice, as well as delivering ACP education (Donnelly et al. 2018; Southern District Health Board 2018; Nevin et al. 2020). In NZ, the importance of education is reflected in the local district health board Service Plan for PC which includes a robust PC education program to support nursing and allied health staff across the two Southern sites and their hospices, through in-person teaching days as well as making resources available for staff and patients (Southern District Health Board 2018).

### Rapid review

The need for rapid review and initiation of care by PC teams is often driven by delays in referrals. Unfortunately, many consultants are reluctant to acknowledge or unable to identify the decline to EOL leading to late referral to the PC team, which in turn leads to insufficient time to assess and follow up, difficulty in making decisions to withdraw care, and support patients in acceptance of a more supportive pathway (Fox et al. 2016; Paes et al. 2018; Southern District Health Board 2018; Nevin et al. 2020; Quinn et al. 2020; Piazza and Drury 2023). Regardless of the increased workload, PC nurses play a crucial role in addressing these challenges associated with late referral, ensuring timely and compassionate care for patients nearing the EOL. The importance of early engagement with PC teams for oncology patients nearing the EOL, allowing sufficient time for review in order to introduce the various community supports available to prevent recurrent ED presentations was highlighted in several studies (Virdun et al. 2016; Southern District Health Board 2018; Chen et al. 2019; Morey et al. 2021; Piazza and Drury 2023). Results from Coombs et al. (2016), concur with these articles, that early assessment, education, and referral to a ward-based PC team, can help reduce unwanted interventions and pointless escalation by acute medical response teams at EOL.

### Bereavement support: An expectation

While bereavement support is recognized as an important aspect of PC, it is often challenging for PC nurses to provide this service due to time constraints and staffing. Nevertheless, this seems to be an expectation that caregivers have of acute PC nurses. In a country where 43% of people die in the acute hospital setting, a study from Ireland has found that bereavement support was lacking in these environments; with areas such as follow-up contact from a counsellor, provision of a memorial service, links to a support group, or financial entitlement needing improvement (Donnelly et al. 2018). Virdun et al. (2016) found that adequate family preparation before death, time to say goodbye, and a system to follow up or assist with bereavement were an expectation of family and caregivers that were not realized. Pre-bereavement interventions and post-death support were important factors for caregivers of patients with advanced illness who had died in acute hospitals, in a cost-effectiveness study into hospital-based specialist PC, where it was also mentioned that hospital resources are scarce and need to be allocated judiciously (Bajwah et al. 2020). The literature, therefore, highlights the discrepancy between the expectations of bereavement support from families and the capacity of acute care PC teams to provide it adequately. The scarcity of resources and the demanding nature of acute care settings often hinder the provision of comprehensive bereavement services, leaving a gap in care for grieving families. The findings highlight the need for increased resources and support for PC nurses to address bereavement needs and ensure a more holistic approach to EOL care that extends beyond the immediate needs of the patient. The importance of such care as part of engagement with patients and their families is reflected in the SDHB service plan, whereby acute needs are attempted to be met in the hospital in a broader holistic manner until other care teams are able to meet these needs (Southern District Health Board 2018). Table 5 summarizes the breadth of roles and responsibilities associated with PC nursing identified in the literature.

**Table 5.** Summary of roles and responsibilities associated with palliative care nursing

Area	Palliative care nursing roles
Coordination of care	Bridging gaps in service delivery, coordinating care across different settings, and facilitating communication between various health care professionals and the patient/family.
Communication and facilitation	Providing clear and compassionate communication to patients, families, and other health care professionals, particularly in complex or emotionally charged situations. Translating medical information and decisions into understandable terms for patients and families. Acting as a liaison and advocate for patients and families, ensuring their needs and preferences are understood and respected.
Discharge planning	Coordinating and facilitating timely and effective discharges, ensuring continuity of care and support for patients transitioning from the hospital to other settings. Collaborating with allied health teams and community services to arrange necessary resources and support for patients and families.
Physical symptom management	Assessing, treating, and managing physical symptoms associated with life-limiting illnesses, utilizing both pharmacological and non-pharmacological interventions. Educating patients and families about symptom management strategies and empowering them to actively participate in their care.
Holistic symptom management	Addressing the psychological, spiritual, social, and existential needs of patients and their families, recognizing the interconnectedness of these dimensions in the experience of suffering. Providing emotional and psychological support, facilitating spiritual care, and addressing social and family concerns. Engaging in pre-bereavement and anticipatory grief work to support patients and families through the emotional challenges of end-of-life care.
Finances	Assisting patients and families in navigating financial concerns related to their illness and care, including accessing funding streams for resources and addressing personal financial challenges that may contribute to existential distress.
Environment	Advocating for and creating a supportive and comfortable environment for patients at the end of life, prioritizing privacy, family involvement, and a sense of peace and dignity. Collaborating with other health care professionals to optimize the physical environment and ensure it contributes to the patient’s overall well-being.
Education	Providing ongoing education to patients, families, and other health care professionals about palliative care, symptom management, advance care planning, and available resources. Promoting understanding and improving the quality of care for patients with life-limiting illnesses.
Rapid review	Conducting timely assessments and initiating care for patients who have experienced a decline or are nearing the end of life, particularly in cases of delayed referrals. Ensuring compassionate and appropriate care is provided promptly, even in the face of increased workload and time constraints.

### Limitations

Studies published prior to 2014 and studies not published in English were excluded. Researcher bias may have influenced the selection of studies and the extraction of data, as the process requires subjective decision-making regarding study inclusion and exclusion criteria. As with all reviews, there is the risk of publication bias, as some research is less likely to be published. The included studies presented data from countries other than New Zealand which may limit the transferability of the findings. In addition, there is limited research involving patient perspectives as PC patients are an extremely vulnerable population. As a result, all available research may not be included in this review; however, the objective is to understand the breadth of available research.

## Conclusion

This scoping review has highlighted the dynamic and often ambiguous nature of PC nursing roles within the acute care setting. The scope of practice extends far beyond the foundational definition of PC, encompassing a diverse array of responsibilities that demand clinical expertise and compassionate care. The increasing complexity of patient needs, coupled with the challenges of coordinating care within an MDT and navigating the often emotionally charged landscape of EOL discussions, results in the demanding nature of this role. The literature reviewed revealed a lack of clarity and consensus regarding PC nurses’ specific roles and expectations, contributing to potential gaps in care and placing significant strain on these health care professionals.
